# Targeting Classical *EGFR* Mutations in Lung Cancer: Twenty Years of Progress and Beyond

**DOI:** 10.3390/cancers18152401

**Published:** 2026-07-25

**Authors:** Yuji Uehara, Hiroto Hatano, Jia-Tao Zhang, Jiefei Han, Kevin L. M. Chua, Stephanie P. L. Saw

**Affiliations:** 1 Department of Thoracic Oncology, National Cancer Center Hospital, Tokyo 1040045, Japan; yuueha2@ncc.go.jp (Y.U.); hihata2@ncc.go.jp (H.H.); 2 Guangdong Lung Cancer Institute, Guangdong Provincial Key Laboratory of Translational Medicine in Lung Cancer, Guangdong Provincial People’s Hospital, Guangdong Academy of Medical Sciences, Southern Medical University, Guangzhou 510030, China; zhangjiatao@gdph.org.cn; 3 Cancer Center, Beijing Chest Hospital, Capital Medical University & Beijing Tuberculosis and Thoracic Tumor Research Institute, Beijing 101149, China; hanjiefei@126.com; 4 Division of Radiation Oncology, National Cancer Centre Singapore, Singapore 168583, Singapore; kevin.chua.l.m@singhealth.com.sg; 5 Division of Medical Oncology, National Cancer Centre Singapore, Singapore 168583, Singapore

**Keywords:** EGFR, NSCLC, osimertinib, amivantamab, lazertinib, adjuvant therapy, neoadjuvant therapy, central nervous system metastases, radiotherapy, resistance, small-cell transformation

## Abstract

The discovery of activating epidermal growth factor receptor (*EGFR*) mutations transformed the management of non-small cell lung cancer and has driven two decades of therapeutic advances in precision oncology across metastatic, resectable, and unresectable locally advanced disease, including central nervous system (CNS) control. Consequently, treatment outcomes for patients with tumors harboring classical *EGFR* mutations have markedly improved. Despite these advances, many questions remain unanswered, and the expanding number of treatment options has created new challenges in treatment selection and sequencing. This narrative review summarizes contemporary data on the management of classical *EGFR*-mutant NSCLC, encompassing novel therapeutic approaches across disease stages, biomarker-informed resistance strategies, multimodality treatment integrating radiotherapy, CNS-directed management, and challenging scenarios such as small-cell transformation. We also discuss emerging fourth-generation inhibitors, nucleic-acid delivery platforms, and data-enabled personalized treatment. We highlight evolving standards of care, unresolved knowledge gaps, and research priorities for the next decade.

## Simple Summary

Classical *EGFR* mutations have transformed the management of non-small cell lung cancer across metastatic, resectable, and unresectable locally advanced settings. This review traces the evolution from early-generation EGFR tyrosine kinase inhibitors to osimertinib and contemporary combination regimens, and summarizes central nervous system management, radiotherapy, acquired resistance, and biomarker-guided treatment. It also addresses unresolved sequencing after FLAURA2 or MARIPOSA, histologic transformation, fourth-generation EGFR inhibitors, nucleic-acid delivery platforms, and data-enabled precision oncology.

## 1. Introduction

**Figure 1:**
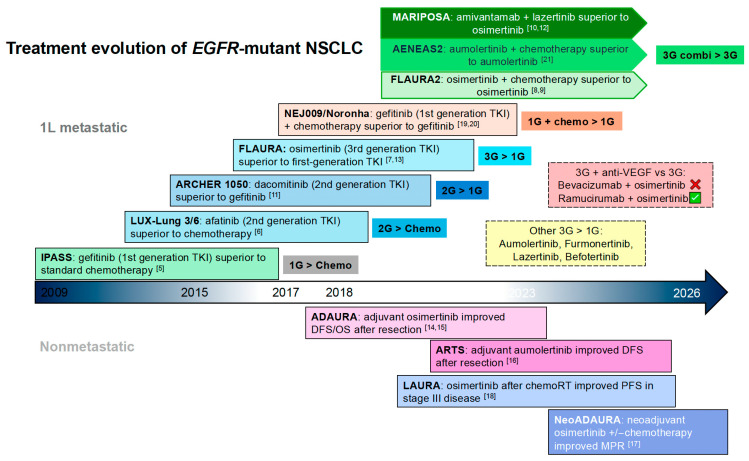
Evolution of therapeutic strategies in classical EGFR-mutant NSCLC. Selected milestones are supported by first-generation EGFR TKI monotherapy and combination trials, second- and third-generation EGFR TKI trials, and studies in resectable, unresectable locally advanced, and metastatic disease [5,6,7,8,9,10,11,12,13,14,15,16,17,18,19,20,21]. Abbreviations: 1G, first-generation; 1L, first-line; 2G, second-generation; 3G, third-generation; chemo, chemotherapy; chemoRT, chemoradiotherapy; DFS, disease-free survival; EGFR, epidermal growth factor receptor; EGFRm, EGFR-mutant; MPR, major pathologic response; NSCLC, non-small cell lung cancer; OS, overall survival; PFS, progression-free survival; RCT, randomized controlled trial; TKI, tyrosine kinase inhibitor; VEGF, vascular endothelial growth factor; vs., versus.

The year 2004 marked a significant milestone in the history of non-small cell lung cancer (NSCLC) with the discovery of activating mutations in the epidermal growth factor receptor (*EGFR*). A subgroup of patients, particularly Asian female never-smokers, were observed to have a dramatic clinical response to gefitinib and were eventually found to have tumors harboring *EGFR* mutations [1,2]. Since then, our understanding of *EGFR*-mutant NSCLC has improved dramatically, from recognizing the various *EGFR* mutation subtypes to understanding treatment resistance and rational combination strategies [3,4]. Figure 1 details the evolution of therapeutic strategies in the first-line metastatic setting for NSCLC harboring classical *EGFR* mutations (exon 19 deletions or L858R point mutations), alongside developments in the non-metastatic setting. Importantly, survival outcomes have improved significantly across all the stages, with *EGFR*-mutant NSCLC representing the poster child for precision medicine in lung cancer. While it has been more than two decades since *EGFR* mutations were first described in lung cancer and treatment options have evolved from first- to third-generation *EGFR* tyrosine kinase inhibitors (TKIs) and from monotherapy to combinatorial approaches, many clinical questions remain unanswered and the promise of cure remains elusive for most patients. Furthermore, as the treatment landscape grows increasingly crowded, other concerns arise, including treatment-related toxicities, financial costs, and the complexity of choosing between multiple therapeutic options—all of which can impact patients’ quality of life.

In this narrative review, we summarize the latest developments in the clinical trial landscape of classical *EGFR*-mutant NSCLC, with emphasis on the swiftly evolving standard(s) of care involving novel compounds and the need for biomarker selection. In addition, we highlight treatment-related paradigms that are shared across early to advanced stage disease, such as the need for continued *EGFR* inhibition, the importance of central nervous system (CNS) protection, risk-stratified approaches, and patient selection factors. Lastly, we discuss challenging clinical scenarios and ongoing controversies that will shape the next decade of progress in *EGFR*-mutant NSCLC.

This narrative review was based primarily on PubMed/MEDLINE searches, supplemented by ClinicalTrials.gov, regulatory and guideline documents, and major oncology meeting reports, including ASCO, ESMO, and WCLC, with the literature updated through 14 July 2026. We prioritized randomized phase II/III trials, pivotal regulatory or guideline-informing studies, and clinically relevant prospective translational studies. Early-phase or preclinical reports were included when required to discuss emerging therapeutic strategies. This review was not designed as a systematic review.

## 2. Treatment Landscape in the Metastatic Setting

### 2.1. First-Line Treatment of EGFR-Mutant NSCLC

The treatment landscape for metastatic *EGFR*-mutant NSCLC has undergone substantial evolution over the past two decades (Table 1). Since the first-generation EGFR TKI gefitinib demonstrated superiority over chemotherapy in the *EGFR*-mutant subgroup of the IPASS trial [5], EGFR TKIs have become the standard treatment for *EGFR*-mutant NSCLC. Subsequently, other EGFR TKIs including erlotinib [22] and second-generation afatinib and dacomitinib [6,11] were developed; however, acquired resistance, particularly mediated by the *EGFR* T790M mutation, remained a major clinical challenge. Osimertinib, a third-generation EGFR TKI, was initially established in T790M-positive disease in AURA3 [23] and later demonstrated superiority over first-generation TKIs in the FLAURA trial [7], while demonstrating a favorable toxicity profile. Since then, osimertinib has become a global standard first-line therapy for *EGFR*-mutant NSCLC. More recently, combination strategies have been developed to further improve outcomes. The FLAURA2 trial evaluated osimertinib in combination with platinum-pemetrexed chemotherapy, demonstrating a significant prolongation in progression-free survival (PFS) and overall survival (OS) compared to osimertinib monotherapy [8,9]. Similarly, the MARIPOSA trial investigated the combination of EGFR-MET bispecific antibody amivantamab and third-generation EGFR TKI lazertinib, demonstrating improved PFS and OS compared with osimertinib alone [10,12]. These studies support first-line treatment intensification as an evidence-based option for appropriately selected patients. Regimen selection should be individualized according to performance status, comorbidities, toxicity tolerance, patient preference, biomarker context, regional approval, drug availability, access, and cost.

The phase III AENEAS2 trial randomized 624 treatment-naive Chinese patients with locally advanced or metastatic *EGFR* exon 19 deletion- or L858R-mutant NSCLC to aumolertinib plus platinum-pemetrexed followed by aumolertinib-pemetrexed maintenance or aumolertinib monotherapy. The combination improved BICR-assessed median PFS (28.9 vs. 18.9 months; HR, 0.47; 95% CI, 0.37–0.60; *p* < 0.0001) and ORR (93.2% vs. 87.3%). OS remained immature (21.6% maturity; HR for death, 0.44; 95% CI, 0.31–0.64), whereas grade ≥3 treatment-emergent adverse events were more frequent with the combination (80% vs. 35%), predominantly reflecting hematologic toxicity [21].

Although upfront combination approaches improve survival, these are invariably at the expense of increased toxicities. The addition of chemotherapy to osimertinib increased the incidence of myelosuppression and gastrointestinal side effects [8,9], whereas amivantamab plus lazertinib resulted in a higher incidence of venous thromboembolism, infusion reactions, and dermatologic toxicity, necessitating prophylactic measures [10,12,24,25]. Given these concerns, a relevant question is whether there are biomarkers that can be used to select patients that benefit more from combination strategies. Co-mutations in tumor suppressor genes (TSGs), particularly *TP53*, have been associated with poorer outcomes in *EGFR*-mutant NSCLC [26,27,28]. The efficacy of treatment intensification in these populations is being evaluated in several clinical trials. The ongoing TOP trial compares osimertinib plus chemotherapy versus osimertinib alone in *EGFR*-mutant NSCLC with concurrent *TP53* mutations, and although the data are still immature, the osimertinib plus chemotherapy group demonstrates improved PFS (median, 34.0 vs. 15.6 months; hazard ratio (HR), 0.44; 95% confidence interval (CI), 0.32–0.61; *p* < 0.001) [29]. The ACROSS2 study compares third-generation EGFR TKI aumolertinib plus chemotherapy versus aumolertinib alone in *EGFR*-mutant NSCLC with concurrent TSG mutations, and shows improved PFS in the aumolertinib plus chemotherapy group (median, 19.8 vs. 16.5 months; HR, 0.58; 95% CI, 0.34–0.97; *p* < 0.05) [30] (Table 1). Both TOP and ACROSS2 suggest that patients with *TP53* co-mutations benefit from upfront chemotherapy added to EGFR TKI, with the caveat that both are China-only studies. It remains uncertain if *TP53* mutations alone are a robust selection biomarker, as this can be confounded by other factors such as tumor burden, and the role of treatment intensification in *TP53*-wildtype tumors should also be evaluated in prospective studies.

**Table 1:** Representative clinical trials involving metastatic *EGFR*-mutant NSCLC in first- and second-line settings.

Study	Phase	Agent	Study Population	Sample Size	ORR (95% CI)	PFS (95% CI)	OS (95% CI)	AE (with 20%+ Incidence) [Grade ≥3]	Ref
**First-line—biomarker-agnostic**									
FLAURA (NCT02296125)	III	Osimertinib	Untreated *EGFR*m (Ex19del/L858R) advanced NSCLC	279	80% (75–85)	18.9 mo (15.2–21.4); HR 0.46 (0.37–0.57); *p* < 0.001	38.6 mo (34.5–41.8); HR 0.80 (0.64–1.00); *p* = 0.046	Diarrhea 60% [3%], Rash/acne 59% [1%], Nail effects 39% [1%], Dry skin 38% [<1%], Stomatitis 29% [<1%], Decreased appetite 24% [3%], Cough 22% [0%], Nausea 20% [0%]	[7,13]
		Gefitinib or Erlotinib		277	76% (70–81)	10.2 mo (9.6–11.1)	31.8 mo (26.6–36.0)	Diarrhea 58% [3%], Rash/acne 79% [7%], Nail effects 34% [1%], Dry skin 37% [1%], ALT increase 27% [8%], AST increase 25% [4%], Stomatitis 22% [<1%], Decreased appetite 21% [2%]	[7,13]
FLAURA2 (NCT04035486)	III	Osimertinib + Platinum + Pemetrexed	Untreated *EGFR*m (Ex19del/L858R) advanced NSCLC	279	83% (78–87)	29.4 mo (25.1–NC); HR 0.62 (0.48–0.80); *p* < 0.001	47.5 mo (41.0–NC); HR 0.77 (0.61–0.96); *p* = 0.02	Anemia 48% [20%], Diarrhea 47% [3%], Nausea 43% [1%], Decreased appetite 33% [3%], Constipation 32% [<1%], Rash 29% [1%], Fatigue 29% [3%], Vomiting 29% [1%], COVID-19 26% [1%], Stomatitis 26% [<1%], Paronychia 25% [1%], Neutropenia 25% [11%], Neutrophil decrease 24% [9%], ALT increase 21% [2%], Dry skin 20% [0%]	[8,9]
		Osimertinib		278	76% (70–80)	16.7 mo (14.1–21.3)	37.6 mo (33.2–43.2)	Diarrhea 42% [<1%], Paronychia 27% [<1%], Dry skin 25% [0%], Rash 22% [0%]	[8,9]
AENEAS2 (NCT04923906)	III	Aumolertinib + Platinum + Pemetrexed	Untreated Chinese EGFRm (Ex19del/L858R) locally advanced or metastatic NSCLC	310	93.2% (89.8–95.8)	28.9 mo (26.3–NE); HR 0.47 (0.37–0.60); *p* < 0.0001	NR (21.6% maturity); HR 0.44 (0.31–0.64)	WBC decrease 87% [34%], Neutrophil decrease 85% [55%], Anemia 76% [11%], ALT increase 71% [7%], AST increase 70% [2%], Platelet decrease 68% [20%], CK increase 56% [10%]	[21]
		Aumolertinib		314	87.3% (83.1–90.7)	18.9 mo (17.8–21.1)	NR	CK increase 51% [9%], AST increase 38% [2%], ALT increase 37% [2%], Weight increase 22% [1%]	[21]
MARIPOSA (NCT04487080)	III	Amivantamab + Lazertinib	Untreated *EGFR*m (Ex19del/L858R) advanced NSCLC	429	86% (83–89)	23.7 mo (19.1–27.7); HR 0.70 (0.58–0.85); *p* < 0.001	NR (42.9-NC); HR 0.75 (0.61–0.92); *p* = 0.005	Paronychia 68% [11%], IRR 63% [6%], Rash 62% [15%], Hypoalbuminemia 48% [5%], ALT increase 36% [5%], Peripheral edema 36% [2%], Constipation 29% [0%], Diarrhea 29% [2%], Stomatitis 29% [1%], AST increase 29% [3%], COVID-19 26% [2%], Anorexia 24% [1%], Pruritus 24% [<1%], Anemia 23% [4%], Nausea 21% [1%], Hypocalcemia 21% [2%]	[10,12]
		Osimertinib		429	85% (81–88)	16.6 mo (14.8–18.5)	36.7 mo (33.4–41.0)	Diarrhea 44% [1%], Rash 31% [1%], Paronychia 28% [<1%], COVID-19 24% [2%], Stomatitis 21% [<1%], Anemia 21% [2%], Cough 21% [0%], Thrombocytopenia 20% [1%]	[10,12]
**First-line—biomarker-selected**									
TOP (NCT04695925)	III	Osimertinib + Platinum + Pemetrexed	Untreated *EGFR*m (Ex19del/L858R) advanced NSCLC with concurrent *TP53* mutation	146	83%	34.0 mo; HR 0.44 (0.32–0.61); *p* < 0.001 (59% maturity)	-	Grade ≥3 TRAEs 62.4% (Details not described)	[29]
		Osimertinib		148	72%	15.6 mo	-	Grade ≥3 TRAEs 14.9% (Details not described)	[29]
ACROSS2 (NCT04500717)	III	Aumolertinib + Platinum + Pemetrexed	Untreated *EGFR*m (Ex19del/L858R) advanced NSCLC with concurrent TSG mutations	62	72% (59–82)	19.8 mo (18.0–NC); HR 0.58 (0.34–0.97); *p* = 0.038	-	Anemia 56% [7%], WBC decrease 50% [11%], AST increase 50% [2%], ALT increase 46% [4%], Neutrophil decrease 43% [17%], Rash 26% [0%], CK increase 22% [2%], Creatinine increase 22% [0%], Platelet decrease 20% [7%], Pruritus 20% [0%], Constipation 20% [0%], Nausea 20% [0%], Hyperuricemia 20% [0%]	[30]
		Aumolertinib		64	67% (55–77)	16.5 mo (12.5–19.2)	-	CK increase 42% [10.9%], AST increase 22% [0%]	[30]
FLOWERS (NCT05163249)	II	Osimertinib + savolitinib	Untreated EGFRm advanced NSCLC with de novo MET amplification or overexpression	21	90.5% (69.6–98.8)	19.6 mo (10.2-NC)	-	Grade ≥3 TRAEs 57.1%	[31]
		Osimertinib		23	60.9% (38.5–80.3)	9.3 mo (7.4-NC)	-	Grade ≥3 TRAEs 8.7%	[31]
**Second-line—biomarker-agnostic**									
MARIPOSA-2 (NCT04988295)	III	Amivantamab + Lazertinib + Chemotherapy	*EGFR*m (Ex19del/L858R) NSCLC after disease progression on osimertinib	263	63% (57–69)	8.3 mo (6.8–9.1); HR 0.44 (0.35–0.56); *p* < 0.001	Not reported at the primary analysis	Neutropenia 69% [55%], Thrombocytopenia 60% [37%], IRR 56% [3%], Anemia 54% [18%], Paronychia 51% [4%], Nausea 50% [6%], Rash 48% [6%], Stomatitis 46% [9%], Leukopenia 40% [27%], Hypoalbuminemia 40% [5%], Constipation 37% [1%], Anorexia 32% [3%], Peripheral edema 32% [0.4%], Vomiting 29% [4%], Fatigue 26% [6%], Diarrhea 26% [4%], Asthenia 25% [5%], Hypokalemia 21% [6%], ALT increase 21% [5%]	[32,33]
		Amivantamab + chemotherapy		131	64% (55–72)	6.3 mo (5.6–8.4); HR 0.48 (0.36–0.64); *p* < 0.001	17.7 mo (16.0–22.4); HR 0.73 (0.54–0.99); *p* = 0.039	IRR 58% [5%], Neutropenia 57% [45%], Nausea 45% [1%], Thrombocytopenia 44% [15%], Rash 43% [6%], Anemia 39% [12%], Constipation 38% [1%], Paronychia 37% [2%], Stomatitis 32% [1%], Peripheral edema 32% [2%], Decreased appetite 31% [0%], Leukopenia 28% [20%], Fatigue 28% [3%], Asthenia 26% [1%], Vomiting 25% [1%], Hypoalbuminemia 22% [2%], COVID-19 21% [2%], ALT increase 20% [5%], Dermatitis acneiform 20% [4%]	[32,33]
		Chemotherapy		263	36% (30–42)	4.2 mo (4.0–4.4)	15.3 mo (13.7–16.8)	Neutropenia 42% [21%], Anemia 40% [9%], Nausea 37% [1%], Thrombocytopenia 30% [9%], Constipation 30% [0%], Leukopenia 28% [9%], ALT increase 28% [4%], AST increase 23% [0%], Anorexia 21% [1%]	[32,33]
OptiTROP-Lung04 (NCT05870319)	III	Sacituzumab Tirumotecan	*EGFR*m (Ex19del/L858R) advanced NSCLC progressed after EGFR TKI	188	61% (53–68)	8.3 mo (6.7–9.9); HR 0.49 (0.39–0.62)	NR (21.5–NE); HR 0.60 (0.44–0.82); *p* = 0.001	Anemia 85% [11%], WBC decrease 84% [28%], Alopecia 84% [0%], Neutropenia 76% [40%], Stomatitis 64% [5%], Nausea 47% [<1%], Anorexia 42% [0%], Fatigue 38% [7%], Weight loss 28% [0%], Thrombocytopenia 27% [4%], Vomiting 27% [0%], ALT increase 24% [<1%], Constipation 21% [0%]	[34]
		Pemetrexed + Platinum		188	43% (36–51)	4.3 mo (4.2–5.5)	17.4 mo (15.7–20.4)	Anemia 76% [14%], WBC decrease 70% [22%], Neutropenia 70% [33%], Nausea 47% [1%], Thrombocytopenia 47% [17%], Fatigue 40% [2%], ALT increase 34% [1%], AST increase 34% [1%], Anorexia 31% [0%], Vomiting 21% [<1%]	[34]
Pooled analysis of TROPION-Lung05 and TROPION-Lung01 (NCT04484142; NCT04656652)	II/III pooled	Datopotamab deruxtecan (Dato-DXd)	*EGFR*m advanced/metastatic NSCLC after *EGFR*-directed therapy and platinum-based chemotherapy	117	43% (34–52)	5.8 mo (5.4–8.2)	15.6 mo (13.1–19.0)	Stomatitis 71% [9%], Nausea 50% [0%], Alopecia 49% [0%], Fatigue 42% [6%], Constipation 31% [0%], Rash 20% [0.8%]. Efficacy: JTO pooled analysis; AE: FDA pooled safety population	[35,36]
HARMONi-A (NCT05184712)	III	Ivonescimab + Pemetrexed + Carboplatin	Advanced *EGFR*m NSCLC post-3rd generation EGFR TKI	219	45%	6.8 mo (5.7–7.1); HR 0.52 (0.41–0.66); *p* < 0.001	16.8 mo (14.3–19.0); HR 0.79 (0.62–1.01); *p* = 0.06	Grade ≥3 TRAEs 50.0% (Details not described)	[37]
		Pemetrexed + Carboplatin		219	34%	4.4 mo (4.1–5.5)	14.0 mo (12.8–15.7)	Grade ≥3 TRAEs 42.2% (Details not described)	[37]
ATTLAS (NCT03991403)	III	Atezolizumab + Bevacizumab + Paclitaxel + Carboplatin	*EGFR*m/*ALK*+ NSCLC post-targeted therapy	154	69.5% (61.5–76.8)	8.48 mo (8.18–10.28); HR 0.62 (0.45–0.86); *p* = 0.004	20.6 mo (18.1–25.6); HR 1.01 (0.69–1.46)	Peripheral neuropathy 72.1% [2.6%], Alopecia 59.6% [0%], Myalgia 27.1% [2.0%]	[38]
		Paclitaxel + Carboplatin		74	41.9% (30.5–53.9)	5.62 mo (4.27–7.2)	20.3 mo (14.3–26.1)	Nausea 23.0% [1.4%]	[38]
ORIENT-31 (NCT03802240)	III	Sintilimab + IBI305 + Pemetrexed + Cisplatin	*EGFR*m NSCLC progressed on EGFR TKI	158	48% (40–56)	7.2 mo (6.6–9.3); HR 0.51 (0.39–0.67); *p* < 0.0001	21.1 mo (17.5–23.9); HR 0.98 (0.72–1.34)	Decreased neutrophil count 66% [20%], Anemia 63% [13%], Decreased white blood cell count 57% [11%], Nausea 53% [3%], Decreased appetite 51% [4%], Asthenia 48% [3%], Increased aspartate aminotransferase 46% [1%], Increased alanine aminotransferase 36% [1%], Vomiting 36% [3%], Decreased platelet count 31% [8%], Hypertension 28% [13%], Increased blood creatinine 25% [0%], Increased gamma-glutamyltransferase 23% [3%], Proteinuria 23% [1%]	[39]
		Sintilimab + Pemetrexed + Cisplatin		158	32% (24–40)	5.5 mo (4.5–6.1); HR 0.72 (0.55–0.94); *p* = 0.02	20.5 mo (15.8–25.3); HR 0.97 (0.71–1.32)	Anemia 67% [8%], Neutropenia 61% [18%], WBC decrease 63% [9%], Nausea 45% [3%], Anorexia 47% [0%], AST increase 42% [0%], Asthenia 40% [1%], ALT increase 38% [0%], Vomiting 24% [1%], Thrombocytopenia 22% [6%], Constipation 20% [0%]	[39]
		Pemetrexed + Cisplatin		160	25% (19–32)	4.3 mo (4.1–5.3)	19.2 mo (15.8–22.4)	Anemia 61% [12%], Neutropenia 54% [19%], WBC decrease 58% [9%], Nausea 49% [1%], Anorexia 43% [1%], AST increase 36% [0%], Vomiting 36% [1%], Asthenia 35% [2%], ALT increase 34% [2%], GGT increase 23% [3%]	[39]
**Second-line—biomarker-informed**									
INSIGHT2 (NCT03940703)	II	Osimertinib + Tepotinib	Post-3G TKI (1L osimertinib) with *MET* amplification	98	50.0% (39.7–60.3)	5.6 mo (95% CI 4.2–8.1)	17.8 mo (95% CI 11.1–NE)	Diarrhea 49% [1%], Peripheral edema 41% [5%], Paronychia 23% [1%], Nausea 21% [2%], Anorexia 20% [4%]	[40]
SAVANNAH (NCT03778229)	II	Osimertinib + Savolitinib	Post-3G TKI with *MET* overexpression/amplification	80	56.3% (44.7–67.3)	7.4 mo (95% CI 5.5–7.6)	NR	Peripheral edema 58.4% [10.9%], Nausea 44.6% [2.0%], Diarrhea 32.7% [3.0%], Vomiting 20.8% [1.0%]	[41]
SACHI (NCT05015608)	III	Osimertinib + Savolitinib	Post-1/2/3G TKI with *MET* amplification	106	58% (49–68)	8.2 mo (6.9–11.2); HR 0.34 (0.23–0.49); *p* < 0.0001	22.9 mo (16.8–NE); HR 0.84 (0.55–1.29)	Leukopenia 49% [7%], Nausea 48% [0%], Vomiting 44% [1%], Anemia 41% [4%], Neutropenia 41% [14%], Hypoalbuminemia 38% [0%], Anorexia 37% [1%], Peripheral edema 36% [0%], Thrombocytopenia 33% [4%], ALT increase 28% [7%], AST increase 28% [6%], Creatinine increase 25% [0%], Pyrexia 24% [1%], Weight loss 23% [0%], Hypocalcemia 22% [1%], Diarrhea 21% [1%], Hypokalemia 21% [3%]	[42]
		Pemetrexed + platinum (cisplatin or carboplatin)		105	34% (25–44)	4.5 mo (3.0–5.4)	17.7 mo (14.9–26.3)	Anemia 73% [23%], Neutropenia 59% [26%], Leukopenia 53% [13%], ALT increase 38% [3%], Nausea 38% [2%], AST increase 36% [0%], Thrombocytopenia 30% [8%], Anorexia 28% [1%], Constipation 24% [1%]	[42]

Abbreviations: 1G, first-generation; 1L, first-line; 2G, second-generation; 3G, third-generation; AE, adverse event; ALK, anaplastic lymphoma kinase; ALT, alanine aminotransferase; AST, aspartate aminotransferase; CI, confidence interval; CK, creatine kinase; COVID-19, coronavirus disease 2019; Dato-DXd, da-topotamab deruxtecan; EGFR, epidermal growth factor receptor; EGFRm, EGFR-mutant; EGFR TKI, EGFR tyrosine kinase inhibitor; Ex19del, exon 19 deletion; FDA, U.S. Food and Drug Administration; GGT, gamma-glutamyl transferase; HR, hazard ratio; IBI305, bevacizumab biosimilar IBI305; IRR, infusion-related reaction; JTO, Journal of Thoracic Oncology; L858R, EGFR L858R mutation; MET, mesenchymal–epithelial transition factor; mo, months; NC, not calculable; NE, not estimable; NR, not reached; NSCLC, non-small cell lung cancer; ORR, objective response rate; OS, overall survival; PFS, progression-free survival; Ref, ref-erence; TKI, tyrosine kinase inhibitor; TRAE, treatment-related adverse event; TSG, tumor suppressor gene; WBC, white blood cell.

### 2.2. Resistance Mechanisms After Third-Generation EGFR TKIs

**Figure 2:**
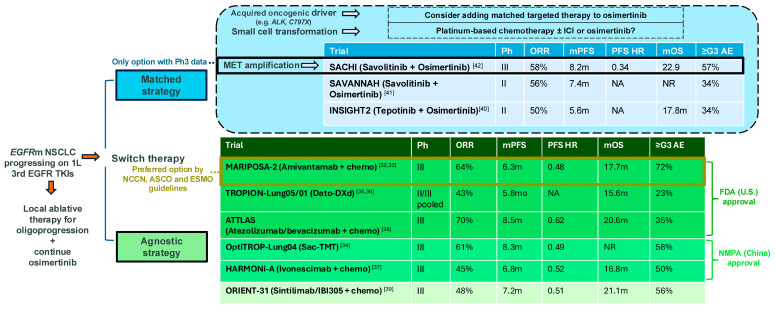
Treatment landscape after progression on first-line osimertinib. Phase II/III biomarker-directed MET-targeted and biomarker-agnostic approaches include MARIPOSA-2, OptiTROP-Lung04, pooled TROPION-Lung05/TROPION-Lung01, HARMONi, ATTLAS, ORIENT-31, INSIGHT2, SAVANNAH, and SACHI [32,33,34,35,36,37,38,39,40,41,42]. Abbreviations: 1L, first-line; AE, adverse event; ALK, anaplastic lymphoma kinase; ASCO, American Society of Clinical Oncology; C797X, EGFR C797X resistance mutation; CNS, central nervous system; EGFR, epidermal growth factor receptor; EGFRm, EGFR-mutant; ESMO, European Society for Medical Oncology; G3, grade 3; HER2, human epidermal growth factor receptor 2; HR, hazard ratio; IBI305, bevacizumab biosimilar IBI305; IC ORR, intracranial objective response rate; ICI, immune checkpoint inhibitor; m, months; MET, mesenchymal–epithelial transition factor; mOS, median overall survival; mPFS, median progression-free survival; NCCN, National Comprehensive Cancer Network; NMPA, National Medical Products Administration; NR, not reached; NSCLC, non-small cell lung cancer; ORR, objective response rate; PFS, progression-free survival; Ph3, phase III; Sac-TMT, sacituzumab tirumotecan; TKI, tyrosine kinase inhibitor.

Despite these advances, acquired resistance inevitably occurs in *EGFR*-mutant NSCLC, and remains a major unmet need (Figure 2). Resistance to osimertinib is mainly mediated by on-target EGFR alterations, off-target bypass pathway alterations or histologic transformation. The most frequent acquired resistance mechanism is *MET* amplification, reported in 17–31% of cases in tissue-based analyses [43,44,45]. Other bypass pathway alterations, such as *CDKN2A/B* deletion and *ERBB2* amplification, have also been described [44,45]. As *EGFR* on-target mechanisms, secondary *EGFR* mutations such as C797X, L718X, and G724X, and *EGFR* amplification occur in approximately 19% and 11%, respectively [44,45]. Histologic transformation reportedly occurs in up to 15% [45,46], with small-cell lung cancer transformation being the most common. Our understanding of resistance mechanisms post-FLAURA2 and MARIPOSA remains limited at present, with only liquid biopsy data available in a subset of patients. Resistance mechanisms appeared similar between osimertinib plus chemotherapy versus osimertinib alone, with *MET* amplification being the most common in both arms and up to two-thirds of patients having no known acquired resistance alteration [47]. In contrast, for MARIPOSA, the frequencies of secondary *EGFR* mutations and *MET* amplification were markedly reduced in the amivantamab plus lazertinib group compared to osimertinib monotherapy, as well as fewer *TP53* mutations and *RB1* loss, suggesting a lower risk of small cell transformation [48]. As the treatment landscape in the first-line setting evolves, a parallel understanding of resistance mechanisms is necessary to guide second-line treatment, and tissue biopsies should be encouraged whenever possible.

**Figure 3:**
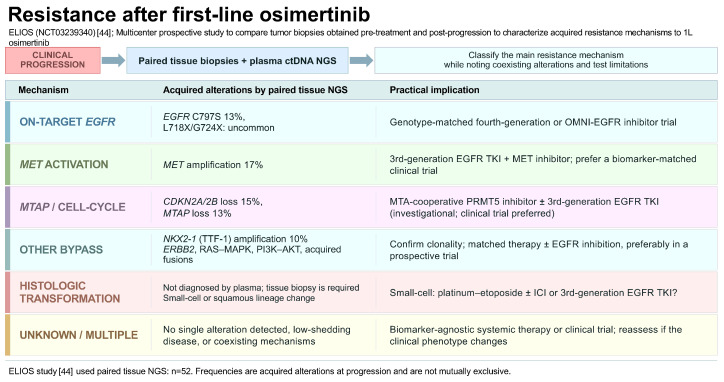
Major resistance mechanisms after third-generation EGFR TKI therapy, selected ELIOS acquired-alteration frequencies, and corresponding treatment strategies. Percentages are from 52 patients with evaluable paired tissue NGS and are not mutually exclusive. *MTAP* loss creates an investigational PRMT5-directed vulnerability; most rare matched strategies remain investigational [40,41,42,43,44,45,46,47,48,49,50,51,52]. Abbreviations: CNS, central nervous system; EGFR, epidermal growth factor receptor; MTA, methylthioadenosine; MTAP, methylthioadenosine phosphorylase; MET, mesenchymal–epithelial transition factor; NGS, next-generation sequencing; NSCLC, non-small cell lung cancer; PRMT5, protein arginine methyltransferase 5; TKI, tyrosine kinase inhibitor.

Recent prospective profiling reinforces both the diversity and coexistence of resistance mechanisms. Among the 52 patients with evaluable paired baseline and post-progression tumor next-generation sequencing in the ELIOS study, acquired MET amplification occurred in 9 (17%), CDKN2A and CDKN2B loss each in 8 (15%), MTAP loss in 7 (13%), and EGFR C797S in 7 (13%) (Figure 3). These alterations were not mutually exclusive [44]. ORCHARD likewise demonstrated heterogeneous and coexisting alterations when tissue and plasma were combined [45].

Clinically relevant categories include on-target *EGFR* alterations (C797X, L718X, G724X, and *EGFR* amplification); bypass signaling through *MET*, *ERBB2*, RAS-MAPK, PI3K-AKT, and acquired kinase fusions; cell-cycle alterations; and small-cell or squamous transformation. Because *MTAP* lies adjacent to *CDKN2A/B* at 9p21.3, homozygous *MTAP* loss frequently co-occurs with *CDKN2A/B* deletion and creates an MTA-dependent vulnerability to PRMT5 inhibition. The MTA-cooperative PRMT5 inhibitor AMG 193 showed preliminary monotherapy activity in *MTAP*-deleted solid tumors, including NSCLC, with an objective response rate of 21% [49,50]. In *MTAP*-deleted *EGFR*-mutant models, BMS-986504 retained activity after TKI exposure and showed additive or synergistic effects with targeted therapy, including tumor control with or without osimertinib in an osimertinib-resistant patient-derived xenograft [51]; related combination studies in other oncogene-driven models support the broader therapeutic principle [52]. These findings support clinical evaluation of a PRMT5 inhibitor plus a third-generation EGFR TKI, but the strategy remains investigational and is not an established treatment (Figure 3).

Plasma genotyping cannot diagnose histologic transformation and may underdetect copy-number changes; tissue-confirmed longitudinal studies are therefore required before plasma observations after FLAURA2 or MARIPOSA can be interpreted as reduced transformation risk. *MTAP* loss should currently be viewed as a co-alteration and therapeutic vulnerability rather than a prospectively validated independent resistance driver.

### 2.3. Treatment After Progression on Osimertinib

Treatment options in the second-line setting have also expanded, resulting in an increasingly crowded therapeutic landscape (Table 1) that can be broadly classified into biomarker-directed and biomarker-agnostic (Figure 2). Among these options, MARIPOSA-2 is the preferred regimen recommended by major guidelines such as the National Comprehensive Cancer Network (NCCN), European Society for Medical Oncology (ESMO), and American Society of Clinical Oncology (ASCO). In the MARIPOSA-2 trial, amivantamab in combination with chemotherapy demonstrated improved PFS compared with chemotherapy alone (median, 6.3 vs. 4.2 months; HR, 0.48; 95% CI, 0.36–0.64; *p* < 0.001) [32]. Importantly, CNS PFS was also improved (median 12.5 months vs. 8.3 months; HR 0.55). The third arm, amivantamab plus lazertinib plus chemotherapy, improved PFS but OS data were not reported at the primary analysis; the regimen was modified during the trial because of increased hematologic toxicity and was not pursued as the preferred combination. TROP2 ADCs have also shown encouraging activity in patients with *EGFR*-mutant NSCLC. The OptiTROP-Lung04 study showed that sacituzumab tirumotecan, a TROP2-directed antibody–drug conjugate (ADC), improves PFS and OS compared with chemotherapy (median PFS, 8.3 vs. 4.3 months; HR, 0.49; 95% CI, 0.39–0.62; median OS, not reached (NR) vs. 17.4 months; HR, 0.60; 95% CI, 0.44–0.82; *p* = 0.001) [34]. Whether these efficacy parameters can be replicated in a global population and represent a new standard of care will be answered in the ongoing TROFUSE-009 study [53]. In a pooled analysis of patients with *EGFR*-mutated NSCLC from TROPION-Lung05/TROPION-Lung01, datopotamab deruxtecan (Dato-DXd), another TROP2-directed ADC, showed clinically meaningful activity after prior targeted therapy and platinum-based chemotherapy (objective response rate (ORR), 43%; median PFS, 5.8 months; median OS, 15.6 months). The US Food and Drug Administration (FDA) has granted accelerated approval for Dato-DXd after prior EGFR-directed therapy and platinum-based chemotherapy based on these results [35,36]. Dual targeting of PD-1 and the vascular endothelial growth factor (VEGF) pathway has also demonstrated improved PFS in several trials; in the HARMONi trial, ivonescimab, a bispecific antibody targeting PD-1 and VEGF, demonstrated improved PFS over chemotherapy [37], while in the ATTLAS study, the addition of bevacizumab and atezolizumab to chemotherapy improved PFS [38]. The ORIENT-31 study investigated the combination of PD-1 inhibitor sintilimab, bevacizumab biosimilar IBI 305, and chemotherapy and demonstrated improved PFS compared with chemotherapy alone [39]. However, no significant OS benefit has been observed in these trials.

While these approaches represent biomarker-agnostic strategies applied across the overall *EGFR*-mutant NSCLC population, biomarker-informed approaches based on resistance mechanisms have recently gained traction. In particular, *MET* amplification or overexpression has emerged as a key therapeutic target, with the added advantage of oral therapeutic options targeting EGFR and MET [54]. The phase III SACHI trial demonstrated that savolitinib plus osimertinib showed a significant improvement in PFS compared with chemotherapy in *EGFR*-mutant NSCLC with acquired *MET* amplification post-TKI (median, 8.2 vs. 4.5 months, HR, 0.34; 95% CI, 0.23–0.49; *p* < 0.0001) [42]. Similarly, the phase II SAVANNAH trial evaluated savolitinib plus osimertinib in *EGFR*-mutant NSCLC with *MET* overexpression or amplification, showing an encouraging ORR of 56.3% [41]. The phase II INSIGHT2 trial also demonstrated promising activity of tepotinib in combination with osimertinib in patients with *MET* amplification, with an ORR of 50.0% [40]. We await the results of the global phase III trial SAFFRON to confirm whether savolitinib plus osimertinib should be the preferred treatment option for acquired *MET* amplification or overexpression post-osimertinib.

### 2.4. Treatment Sequencing After Contemporary First-Line Regimens

**Figure 4:**
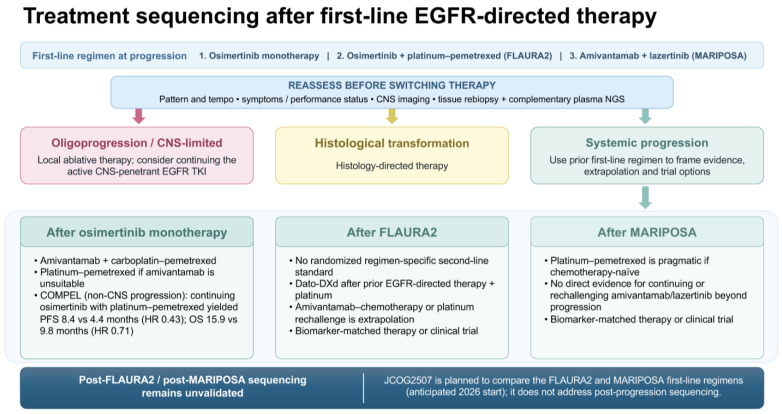
Practical treatment sequencing after progression on contemporary first-line regimens for classical EGFR-mutant NSCLC. The algorithm distinguishes oligoprogression from systemic progression, incorporates COMPEL for selected non-CNS progression after first-line osimertinib, and separates evidence-supported treatment from extrapolation or trial-only strategies. No prospectively validated post-progression sequence exists after FLAURA2 or MARIPOSA; JCOG2507 is planned to compare the two first-line regimens [31,32,33,35,36,40,41,42,44,45,55,56,57,58,59,60]. Abbreviations: CNS, central nervous system; COMPEL, osimertinib plus platinum-pemetrexed beyond non-CNS progression; Dato-DXd, datopotamab deruxtecan; EGFR, epidermal growth factor receptor; FLAURA2, osimertinib plus platinum-pemetrexed; HR, hazard ratio; JCOG, Japan Clinical Oncology Group; MARIPOSA, amivantamab plus lazertinib; NGS, next-generation sequencing; OS, overall survival; PFS, progression-free survival; TKI, tyrosine kinase inhibitor.

At progression, the first step is to distinguish oligoprogression or isolated CNS progression from systemic progression and to reassess histology and molecular resistance (Figure 4). Tissue biopsy is preferred when feasible because it can identify transformation, copy-number changes, and protein expression; plasma next-generation sequencing is complementary and may better capture spatial heterogeneity. A negative plasma result should prompt tissue testing when clinically feasible, particularly for CNS-predominant or low-shedding disease.

After osimertinib monotherapy, local ablative therapy with continued CNS-active EGFR inhibition may be considered for selected oligoprogression. In COMPEL, 98 patients with non-CNS progression after first-line osimertinib were randomly assigned to continue osimertinib or receive placebo with platinum-pemetrexed. Continuing osimertinib was associated with a median PFS of 8.4 versus 4.4 months (HR, 0.43; 95% CI, 0.27–0.70) and a numerically longer median OS of 15.9 versus 9.8 months (HR, 0.71; 95% CI, 0.42–1.23) [55]. Because recruitment was curtailed and efficacy analyses were descriptive, these results support consideration in selected patients with non-CNS progression but do not establish a universal post-osimertinib standard.

For patients treated with upfront combination therapy (FLAURA2 or MARIPOSA regimen), the optimum next-line treatment remains controversial. As datopotamab deruxtecan has been FDA-approved for patients who have received prior *EGFR*-directed therapy and platinum-based chemotherapy, this is a potential option for patients who have progressed on FLAURA2, although the data supporting this approach are limited to a retrospective pooled analysis from two clinical trials [35,36]. The efficacy of alternative approaches after FLAURA2, such as platinum-pemetrexed rechallenge or amivantamab plus chemotherapy for patients progressing on maintenance osimertinib, remains to be validated. For patients treated with MARIPOSA, even less is known about what should be done after progression, including whether amivantamab or lazertinib should be continued for *EGFR* inhibition.

Across both combination regimens, biomarker-matched therapy, histology-directed platinum-etoposide for small-cell transformation, and clinical trials should be prioritized when appropriate. No completed head-to-head trial has compared the FLAURA2 and MARIPOSA regimens, and no prospective study has defined the optimal post-progression sequence after either combination. The JCOG Lung Cancer Study Group plans JCOG2507, a comparison of osimertinib plus platinum-based chemotherapy (FLAURA2) versus amivantamab plus lazertinib (MARIPOSA) in the first-line setting, with enrollment expected to begin in 2026 [56]; however, this trial addresses first-line regimen selection rather than post-progression sequencing (Figure 4).

With the increasing diversity of treatment options for *EGFR*-mutant NSCLC, clinical practice is shifting towards more personalized treatment strategies. In particular, biomarker-guided understanding of resistance mechanisms in individual patients is critical for optimal treatment selection [57,58].

In understanding resistance mechanisms, tissue biopsy remains the gold standard, as it enables comprehensive evaluation of genomic alterations and protein-level changes. However, tissue biopsies carry a risk of procedure-related complications, and not all patients have accessible target lesions upon progression. In addition, single-site sampling often fails to capture intratumoral heterogeneity. In this context, liquid biopsy has emerged as a potential alternative. Analysis of circulating tumor DNA (ctDNA) can detect genomic alterations and capture spatial tumor heterogeneity. However, its sensitivity remains limited; for example, the sensitivity for detecting *MET* amplification has been reported to be as low as 15–23% [43,59] compared with tissue-based assays. Additionally, protein-level alterations or histological transformation cannot be detected by ctDNA. While novel sequencing technologies have shown promise for assessing protein expression on circulating tumor cells, current evidence is limited to small studies [60], and their utility in guiding treatment selection requires further validation.

Appropriate patient selection is also essential when performing re-biopsy and subsequent molecular testing to elucidate resistance mechanisms. These analyses require a certain turnaround time, which may not be feasible in patients with rapidly progressive disease. Additionally, post-osimertinib resistance mechanisms are diverse, and even the most common and druggable *MET* amplification is detected in only 17–31% of cases [43,44,45], suggesting that a repeat biopsy may not change treatment recommendations for some patients. Collectively, these challenges highlight the need for careful patient selection for biomarker-guided approaches.

Finally, treatment sequencing remains an important unresolved issue. Although the MET pathway represents a key resistance mechanism, it remains unclear whether it should be targeted upfront in the first-line setting, such as using amivantamab plus lazertinib, or reserved for later lines of therapy in the context of acquired MET resistance. The randomized phase II FLOWERS study of first-line osimertinib with or without savolitinib in *MET*-aberrant *EGFR*-mutant NSCLC suggests that the addition of MET TKI to osimertinib upfront may benefit patients with de novo *MET* aberrations [31], but the broader sequencing question remains unresolved. Moreover, treatment intensification strategies in the first-line setting are associated with increased toxicity, underscoring the importance of shared decision-making with patients.

## 3. EGFR TKI in Resectable Disease

The standard of care for resectable *EGFR*-mutant NSCLC has been profoundly reshaped by the success of targeted therapy in the adjuvant setting. However, as we move beyond the landmark ADAURA study [14,15], a new series of critical questions is emerging regarding the role of chemotherapy, the goal of adjuvant treatment, the value of neoadjuvant EGFR TKI, and the integration of molecular residual disease (MRD). The major neoadjuvant/peri-operative and adjuvant studies are summarized in Table 2 and Table 3.

### 3.1. Current Controversies: The Role of Chemotherapy and the Potential for Cure

Spanning more than two decades of clinical investigation, the adjuvant EGFR TKI journey has progressed from unselected trials (BR.19 [61], RADIANT [62]) to first-generation TKI studies with limited disease-free survival (DFS) benefit (CTONG1104 [63], IMPACT [64]) and consistent failure to improve OS, culminating in the phase III ADAURA trial [14], in which third-generation osimertinib achieved a significant DFS and OS advantage (Table 2). ARTS, which adopted a similar study design to ADAURA, also reported consistent findings, although OS remains immature [16]. Some possible explanations to account for the differences in trial results comparing first-generation and third-generation adjuvant EGFR TKI trials include the duration of EGFR TKI (two versus three years), with adjuvant chemotherapy being the comparator arm in first-generation EGFR TKI trials, whereas it was allowed in both arms of ADAURA and ARTS, as well as the superior intracranial efficacy of third-generation EGFR TKI that contributed to improved CNS PFS [65]. These advances notwithstanding, it remains debatable whether adjuvant chemotherapy is still necessary in the era of third-generation EGFR TKIs. While ADAURA’s subgroup analysis showed consistent DFS and OS benefits for osimertinib regardless of prior adjuvant chemotherapy, the trial was not designed to test this comparison [66]. Notably, exploratory analyses from the ADAURA trial revealed that patients with stage II-IIIA disease who received adjuvant chemotherapy in addition to osimertinib had a 5-year OS of 87%, whereas those who received adjuvant osimertinib without chemotherapy had a 5-year OS of 80%, although formal statistical testing was not performed [15]. Therefore, the current evidence does not permit a definitive answer to this question. OCEANiC (ACTRN12623000552684) [67] is an ongoing phase II study by the TOGA group that is examining the benefit of adjuvant osimertinib alone for patients with negative ctDNA and co-mutation results, whereas patients with positive ctDNA and/or co-mutation-positive disease will receive adjuvant chemotherapy followed by osimertinib. However, as this is a non-randomized study with no control arm, whether the results will be sufficient to change clinical practice remains an open question. To that end, in the absence of randomized data, should adjuvant chemotherapy be discussed with otherwise fit patients with stage II-IIIA disease, taking into consideration patients’ preferences and treatment tolerability? Beyond statistical limitations, patient preferences further complicate the picture. As Wu and colleagues noted in their editorial accompanying the FLAURA2 trial, a survey revealed that 73% of patients with lung cancer preferred monotherapy over combination therapy, even when told that combination therapy could delay disease progression by 9 months—a powerful reflection of the fear of chemotherapy-related toxicity [68].

**Table 2:** Adjuvant studies of EGFR TKI in resected EGFR-mutant NSCLC.

Study NCT	Phase	Population	Intervention vs. Control	Primary Endpoint	DFS/EFS	OS	Ref
**Completed—Phase III**							
ARTS (NCT04687241)	III	Stage II–IIIB, *EGFR*m (Ex19del/L858R), n = 204	Aumolertinib 110 mg QD × 3 y vs. Placebo	DFS	mDFS NR vs. 24.9 m; HR = 0.17 (0.08–0.33); *p* < 0.0001 2-y DFS 88.2% vs. 53.2%	Immature (HR not reported)	[16]
ADAURA (NCT02511106)	III	Stage IB–IIIA (8th AJCC), *EGFR*m (Ex19del/L858R), n = 682	Osimertinib 80 mg QD × 3 y vs. Placebo	DFS (II–IIIA); DFS (IB) key secondary	Stage II–IIIA: mDFS 65.8 vs. 21.9 m; HR = 0.23 (0.18–0.30); *p* < 0.001; 4-y DFS 70% vs. 29% Stage IB: HR = 0.39 (0.26–0.58); *p* < 0.001; 4-y DFS 80% vs. 73%	5-y OS (II–IIIA): 85% vs. 73%; HR = 0.49 (0.34–0.71); *p* < 0.001 Stage IB OS: HR = 0.57 (0.33–0.97); *p* = 0.032	[14,15]
**Ongoing—Phase II/III**							
TARGET (NCT05526755)	II	Stage II–IIIB, *EGFR*m (incl. uncommon), n = 188 (planned)	Osimertinib 80 mg QD × 5y	5-y DFS rate	-	-	[69]
ADAURA2 (NCT05120349)	III	Stage IA2–IA3, *EGFR*m (Ex19del/L858R), n≈300	Osimertinib 80 mg QD × 3 y vs. Placebo	DFS	-	-	[70]
**Completed—Phase II/III**							
CORIN/GASTO1003 (NCT02513655)	II	Stage IB–IIIA, *EGFR*m, n = 128	Icotinib 125 mg TDS (12 m) vs. Observation	3-y DFS	3-y DFS 73.4% vs. 45.3%; HR = 0.20 (0.08–0.47); *p* = 0.0003 5-y DFS 67.3% vs. 44.1%	5-y OS 93.3% vs. 76.7% (HR not reported)	[71,72]
IMPACT JPRN-UMIN000003830	III	Stage II–III, *EGFR*m, n = 234	Gefitinib 250 mg QD (2 y) vs. Cis/Vin	DFS	mDFS 35.9 vs. 25.1 m; HR = 0.92 (0.66–1.28); *p* = 0.63	HR = 1.03 (0.70–1.52); *p* = 0.87	[64]
EVIDENCE (NCT02448762)	III	Stage II–IIIA, *EGFR*m, n = 322	Icotinib 125 mg TDS (12 m) vs. Cis/Vin	DFS	mDFS 47.0 vs. 22.1 m; HR = 0.36 (0.24–0.55); *p* < 0.0001 3-y DFS 63.9% vs. 32.5%	HR = 0.86 (0.52–1.42); *p* = 0.556 (NS) at IA	[73]
ADJUVANT/CTONG1104 (NCT01405079)	III	Stage II–IIIA (N1/N2), *EGFR*m, n = 222	Gefitinib 250 mg QD (2 y) vs. Cis/Vin	DFS	mDFS 28.7 vs. 18.0 m; HR = 0.60 (0.42–0.86); *p* = 0.005 3-y DFS 39.6% vs. 32.5%	HR = 0.92 (0.62–1.36); *p* = 0.674 (NS); mOS 75.5 vs. 62.8 m	[63,74]
SELECT (NCT00567359)	II	Stage IA–IIIA, *EGFR*m, n = 100	Erlotinib 150 mg QD (2 y)	2-y DFS rate	2-y DFS 88% (vs. 76% historical); 5-y DFS 56%; 5-y OS 86%	5-y OS 86%	[75]
EVAN (NCT00660287)	II	Stage IIIA (N2), *EGFR*m, n = 102	Erlotinib 150 mg QD (2 y) vs. Cis/Vin	2-y DFS rate	2-y DFS 81.8% vs. 44.6%; HR = 0.27 (0.13–0.53); *p* < 0.001 5-y DFS 51.5% vs. 25.2%	mOS 84.2 vs. 61.1 m; HR = 0.46 (0.23–0.93); *p* = 0.029 5-y OS 66.1% vs. 59.7%	[76,77]
RADIANT (NCT00339183)	III	Stage IB–IIIA, *EGFR* IHC+, n = 973	Erlotinib 150 mg QD (2 y) vs. Placebo	DFS (IHC+ primary)	HR = 0.81 (0.65–1.00); *p* = 0.039 (not significant for primary ITT) 4-y DFS 46.4% vs. 41.6%	HR = 0.99 (0.79–1.25)	[62]
BR.19 (NCT00329654)	III	Stage IB–IIIA, unselected for *EGFR*, n = 503	Gefitinib 250 mg QD (2 y) vs. Placebo	OS	HR = 1.22 (0.93–1.61)	HR = 1.24 (0.94–1.64); *p* = 0.14	[61]

**Abbreviations**: AJCC, American Joint Committee on Cancer; CI, confidence interval; DFS, disease-free survival; EFS, event-free survival; EGFR, epidermal growth factor receptor; EGFRm, EGFR-mutant; Ex19del, exon 19 deletion; HR, hazard ratio; IA, interim analysis; IHC, immunohistochemistry; ITT, intention-to-treat; L858R, EGFR L858R mutation; m, months; mDFS, median disease-free survival; NCT, ClinicalTrials.gov identifier; NR, not reached; NSCLC, non-small cell lung cancer; OS, overall survival; QD, once daily; Ref, reference; TKI, tyrosine kinase inhibitor; vs., versus; y, years.

A second unresolved question is whether adjuvant TKI offers a true cure or merely delays disease recurrence. The convergence of DFS curves after TKI cessation was first observed in the first-generation EGFR TKI trials, which consequently did not translate into a significant OS benefit. Similarly, although the ADAURA trial demonstrated a statistically robust 10% absolute improvement in 5-year OS with adjuvant osimertinib [15], the observation that 58% of recurrences or MRD-detected molecular relapses occurred within the first year after cessation of TKI therapy suggests that the benefit may reflect disease suppression rather than eradication [78]. While a 3-year course of osimertinib may be insufficient for these patients, it is equally important to recognize that up to 40% of patients with resected stage IB-IIIA *EGFR*-mutant remain disease-free at five years even without adjuvant osimertinib [79], underscoring the importance of risk-stratified approaches. Ongoing trials include TARGET [69], which evaluates 5 years of adjuvant osimertinib and includes a cohort for uncommon mutations (G719X, S768I, L861Q). ADAURA2 [70] and APPOINT [80] are both evaluating the benefit of adjuvant osimertinib and aumolertinib, respectively, in stage IA disease (Table 2). We await future trials to inform the optimal duration of adjuvant EGFR TKI and to guide individualized treatment decisions.

### 3.2. Neoadjuvant EGFR TKI: A Modest Beginning

The enthusiasm generated by adjuvant osimertinib, however, has not been mirrored in the neoadjuvant setting. Table 3 summarizes the key neoadjuvant and peri-operative trials for *EGFR*-mutant NSCLC. In the phase IIb NEOS trial [81], neoadjuvant osimertinib monotherapy achieved an ORR of 71% but a major pathological response (MPR) rate of only 11%. Subsequently, the phase III NeoADAURA trial explored peri-operative osimertinib with or without chemotherapy; pathologic complete response (pCR) was 4% with osimertinib alone, 9% with osimertinib plus chemotherapy, and 0% with placebo plus chemotherapy, while MPR rates were 26%, 25%, and 2%, respectively [17]. Importantly, the added benefit of neoadjuvant osimertinib remains uncertain, especially given that adjuvant osimertinib was administered to 91% of patients across all three arms, and long-term survival outcomes are awaited. Two phase II trials involving neoadjuvant osimertinib given over 6–8 weeks similarly observed limited pathological responses, with both trials achieving 0% pCR and MPR rates of 15% and 24%, respectively [82,83].

Given the high ORRs achieved with EGFR TKIs, neoadjuvant TKI therapy holds particular promise in converting initially unresectable disease to operable status. The phase II LungMate 007 trial of neoadjuvant aumolertinib in 51 patients with initially unresectable stage III *EGFR*-mutant NSCLC reported an ORR of 70.6% and a surgical conversion rate of 45%, with all converted patients achieving R0 resection [84]. Meanwhile, the ongoing phase III APPROACH/CTONG2101 trial is also evaluating this conversion paradigm [85]. In this study, patients with unresectable stage III *EGFR*-mutant NSCLC receive 8 weeks of aumolertinib induction therapy, followed by MDT evaluation to determine subsequent local treatment. APPROACH aims to validate the feasibility of converting initially unresectable disease to operable status. Thus, despite the modest pathological responses observed in the resectable setting as detailed above, induction or neoadjuvant TKI therapy may represent a promising strategy for converting initially unresectable *EGFR*-mutant NSCLC to resectable disease, and ongoing trials are highly anticipated.

**Table 3:** Neoadjuvant and peri-operative studies of EGFR TKI or EGFR-directed therapy in resectable NSCLC.

Study NCT	Phase	Setting	Population	Intervention vs. Control	Primary Endpoint	pCR	MPR	EFS	ORR	OS	Ref
**Completed—Phase III**											
NeoADAURA (NCT04351555)	III	Peri-operative	Stage II–IIIB, EGFRm (Ex19del/L858R), n = 358	Arm A: osimertinib 80 mg QD (neoadj → adj 3 y)	MPR	4%	26%	vs. Arm C: HR 0.48 (0.27–0.86)	-	NR	[17]
				Arm B: osimertinib + chemotherapy (neoadj → adj 3 y)	MPR	9%	25%	vs. Arm C: HR 0.53 (0.31–0.90); vs. Arm A: NS	-	NR	[17]
				Arm C: placebo + chemotherapy (neoadj → adj 3 y)	MPR	0%	2%	Reference	-	NR	[17]
**Completed—Phase II**											
NORA (NCT04816838)	II	Peri-operative	Stage I–IIIA, *EGFR*m, n = 25	Osi 8 wks pre-op → Surgery → Osi adj 3 y	ORR	0%	24%	-	44%	NR	[83]
NCT03433469	II	Neoadjuvant	Stage I–IIIA, *EGFR*m, n = 27	Osimertinib 80 mg QD for up to two 28-day cycles preoperatively	MPR (≥40%)	0%	14.8% (95% CI 4.2–33.7)	-	51.9%	NR	[82]
NEOS (NCT02840076)	IIb	Neoadjuvant	Stage IIA–IIIB (T3–4N2), *EGFR*m, n = 40	Osimertinib 80 mg QD (6 wks pre-op)	ORR	3.6%	10.7%	-	71.1% (95% CI 54.1–84.6)	NR	[81]
EMERGING/CTONG1103 (NCT01407822)	II	Neoadjuvant	Stage IIIA-N2, *EGFR*m, n = 72	Erlotinib 150 mg QD (42d) vs. Gem/Cis	ORR	-	-	HR = 0.39 (0.22–0.68) mPFS 21.5 vs. 11.4 m	54.1% vs. 34.3% OR = 2.26 (0.95–5.44)	No OS benefit (HR = 1.24; *p* = 0.44)	[86]
**Ongoing—Phase II**											
ANSWER NCT04455594	II	Neoadjuvant	Stage IIIA-N2, *EGFR*m, n = 168 (planned)	Aumolertinib vs. Erlotinib/Chemo	ORR	-	-	-	Ongoing	-	[87]
Neoafa NCT04470076	II	Neoadjuvant	Stage IIA–IIIB, *EGFR*m (incl. uncommon), n = 30	Afatinib + Pem/Carbo (~12 wks)	MPR, ORR	-	-	-	-	-	[88]
NEOpredict-EGFR NCT06784791	II	Neoadjuvant	Stage IB–IIIA, *EGFR*m (incl. exon 20), n = 20	Amivantamab ± Carbo/Pem (4–12 wks)	Feasibility (R0 rate)	-	-	-	-	-	[89]

**Abbreviations:** adj, adjuvant; AE, adverse event; chemo, chemotherapy; CI, confidence interval; EFS, event-free survival; EGFR, epidermal growth factor receptor; EGFRm, EGFR-mutant; Ex19del, exon 19 deletion; HR, hazard ratio; MPR, major pathologic response; NCT, ClinicalTrials.gov identifier; neoadj, neoadjuvant; NR, not reached; NS, not significant; NSCLC, non-small cell lung cancer; ORR, objective response rate; OS, overall survival; Osi, osimertinib; pCR, pathologic complete response; pre-op, preoperative; QD, once daily; R0, microscopically margin-negative resection; Ref, reference; vs., versus; wks, weeks; y, years.

### 3.3. MRD-Guided Adjuvant Therapy: Opportunities and Challenges

Minimal residual disease (MRD) is an attractive concept for personalizing adjuvant therapy decisions, particularly regarding the duration of EGFR TKI. In a prospective observational study of 71 patients with resected stage I-III *EGFR*-mutant NSCLC, 22 patients who suspended adjuvant TKI based on sustained undetectable MRD achieved a 2-year DFS rate of 80%, with median DFS not reached [90]. Several ongoing studies are prospectively evaluating MRD-guided strategies, including APPROACH/CTONG2101 in unresectable stage III disease [85] and NCT05536505 and CTONG2201 in the adjuvant setting [91,92].

However, it is also pertinent to consider that MRD monitoring in *EGFR*-mutant NSCLC faces unique challenges, including low ctDNA shedding, brain metastasis coverage, and panel selection [93,94]. The DRIVE study demonstrated that driver-focused MRD assays achieve prognostic performance comparable to tumor-informed or large-panel approaches, with superior risk discrimination in certain subsets [95]. Cost-effectiveness and turnaround times are important metrics to capture in MRD-guided approaches, as repeated longitudinal MRD testing may increase the financial burden for patients, and delayed results may impact treatment decisions. Furthermore, whether MRD should be used to de-escalate and/or escalate treatment strategies remains an open question, for which dedicated trials are needed to definitively answer [96].

## 4. Management of CNS Disease in EGFR-Mutant NSCLC

Central nervous system metastases represent the most devastating complication of *EGFR*-mutant NSCLC. Brain parenchymal metastases (BM) and leptomeningeal metastases (LM) are the two predominant forms of intracranial dissemination, with distinct biological behaviors and clinical outcomes [97].

### 4.1. Epidemiology and Diagnosis

BM occurs in 20–40% of patients with advanced NSCLC, whereas LM is less frequent but far more lethal [98,99]. The cumulative incidence of CNS metastases in *EGFR*-mutant NSCLC increases progressively from 34% at 1 year to 53% at 5 years [100]. While LM affects only 3–5% of unselected NSCLC patients, its incidence rises to 11% in *EGFR*-mutant cases [101], and it confers a dismal prognosis with a median OS of just 3.6–11 months in historical cohorts, compared to 28.9 months for BM [99,102,103,104].

Clinically, BM typically presents as discrete gray-white matter junction lesions causing focal neurological deficits, seizures, or increased intracranial pressure [105]. In contrast, LM manifests as diffuse leptomeningeal infiltration, leading to multifocal symptoms including cranial neuropathies, gait instability, and cognitive impairment [98,106].

Contrast-enhanced magnetic resonance imaging (MRI) remains the primary diagnostic modality for both forms of CNS metastases [107]. While MRI has high sensitivity for BM, its sensitivity for LM is only 60–70% [108]. Advanced sequences, such as three-dimensional contrast-enhanced T1-SPACE and contrast-enhanced fluid-attenuated inversion recovery (CE-FLAIR), have significantly improved the detection of subtle leptomeningeal enhancement [109].

### 4.2. CNS Efficacy of Standard First-Line Therapies

Third-generation *EGFR* TKIs have transformed the management of CNS metastases in *EGFR*-mutant NSCLC. Osimertinib, the current standard first-line therapy, demonstrates superior blood–brain barrier (BBB) penetration with a cerebrospinal fluid (CSF) to plasma ratio of 21% [110,111]. In the landmark FLAURA study, osimertinib achieved an intracranial objective response rate (iORR) of 91% and a median intracranial progression-free survival (iPFS) of 15.2 months, significantly outperforming first-generation TKIs [7,112].

Building on this foundation, the FLAURA2 study investigated osimertinib plus platinum-pemetrexed chemotherapy in 557 treatment-naive patients, 40% of whom had baseline CNS metastases. In the CNS efficacy analysis, osimertinib plus chemotherapy improved CNS PFS compared with osimertinib alone (median, 30.2 vs. 27.6 months; HR, 0.58; 95% CI, 0.33–1.01), highlighting that upfront treatment intensification improves CNS outcomes in addition to PFS and OS [113].

More recently, the MARIPOSA study evaluated amivantamab plus lazertinib versus osimertinib monotherapy. In the pre-specified CNS subgroup analysis of 264 patients with baseline metastases, the combination regimen showed numerically longer intracranial PFS: median iPFS reached 25.4 months versus 22.2 months with osimertinib alone (HR 0.79, 95% CI 0.61–1.02), and iORR was 78% versus 77% [12]. Notably, as a large-molecule monoclonal antibody, amivantamab has limited BBB penetration, and its intracranial activity may rely heavily on lazertinib. More data are needed to understand the activity of amivantamab within the CNS.

### 4.3. Intracranial Activity of Novel Therapies

For patients who develop resistance to first-line EGFR TKI regimens, novel therapeutic classes are providing new options for CNS disease. ADCs have emerged as a particularly exciting class, with preliminary data demonstrating intracranial efficacy [114]. Patritumab deruxtecan (HER3-DXd) achieved an iORR of 33.3% in patients with BM and demonstrated preliminary activity in LM in the TUXEDO-3 trial [115,116]. Datopotamab deruxtecan (Dato-DXd), a TROP2-directed ADC, is also under active investigation for both BM and LM [117]. While sacituzumab tirumotecan has demonstrated significant survival benefit over platinum-pemetrexed chemotherapy in OptiTROP-Lung04, its CNS efficacy has not been reported. Additionally, the combination of amivantamab and lazertinib has shown encouraging intracranial activity in osimertinib-resistant patients [32]. However, these datasets remain limited and may also be confounded by previous brain radiotherapy. The optimal role of these novel agents, either alone or in combination with EGFR TKIs, remains to be fully defined and requires careful balancing of efficacy benefits against potential increased toxicity risks.

### 4.4. Intrathecal Therapy and CSF Liquid Biopsy

Despite advances in systemic therapy, LM remains uniquely challenging due to the limited ability of most systemic agents to cross the intact blood–CSF barrier (BCSFB) [104]. For patients with *EGFR* TKI-refractory LM, intrathecal pemetrexed has emerged as a promising option, although data are limited to phase II trials. In the largest prospective phase II trial to date (n = 132) [102], intrathecal pemetrexed achieved a Response Assessment in Neuro-Oncology (RANO)-assessed response rate of 80% and a median OS of 12.0 months, with manageable toxicity. Notably, treatment-induced CSF cytological clearance was associated with a dramatic survival benefit (median OS 16.0 months versus 4.0 months, *p* < 0.001). Importantly, intrathecal pemetrexed remains effective even in patients with poor performance status (Eastern Cooperative Oncology Group [ECOG] 3–4) and those who have failed prior intrathecal methotrexate or cytarabine therapy [104].

Accurate diagnosis and monitoring of LM depend critically on CSF analysis. Although CSF cytology is the historical gold standard, its limited sensitivity (approximately 50–90% with a single lumbar puncture) necessitates repeated sampling [104]. In recent years, CSF circulating tumor DNA (ctDNA) profiling has emerged as a superior diagnostic tool, with detection rates of 85% in LM patients and 46% in BM patients, including 73% of cytology-negative LM cases [118].

Beyond diagnosis, CSF ctDNA provides critical prognostic and therapeutic information. Detectable CSF ctDNA is an independent adverse prognostic factor, associated with nearly two-fold higher mortality [118]. Compared to plasma ctDNA, CSF ctDNA consistently demonstrates higher variant allele frequencies and superior detection sensitivity for intracranial disease [119]. Most importantly, it frequently identifies compartment-specific molecular alterations and resistance mechanisms—including *EGFR* C797S mutations, *MET* amplification, and *ERBB2* activation—that are undetectable in matched plasma or tissue samples, highlighting the unique molecular evolution of CNS metastases [118]. Longitudinal CSF ctDNA monitoring can also detect early CNS progression and guide timely therapeutic adjustments. While not yet universally standard, CSF ctDNA has revolutionized LM management and is rapidly becoming an essential tool for guiding therapeutic decisions in *EGFR*-mutant NSCLC with CNS metastases.

## 5. Radiotherapy in Locally Advanced and Metastatic Disease

A central challenge in *EGFR*-mutated NSCLC is defining how best to combine highly effective targeted therapy with the cytoreductive efficacy of modern precision radiotherapy (RT).

### 5.1. Reconsidering Upfront RT in Brain Metastases

The combined STARLET analysis of LUOSICNS and OUTRUN suggests that upfront RT may be deferred when frontline osimertinib is used [120]. With earlier-generation TKIs, randomized data similarly support possible deferral only in carefully selected patients with asymptomatic, limited or non-bulky CNS disease, no indication for urgent local control, and access to close serial brain MRI surveillance and prompt salvage therapy if needed [121]. Ongoing studies will evaluate whether certain subgroups of patients may benefit from upfront brain radiotherapy—potentially patients with oligometastatic disease [122] or patients with bulky brain metastases [123].

### 5.2. Establishing the Role of EGFR TKIs in Stage III Unresectable Disease

In Stage III unresectable NSCLC, early attempts to combine gefitinib with RT were disappointing in the unselected population [124]. In *EGFR*-mutated Stage III disease, gefitinib given concurrently with and after thoracic RT was feasible and showed activity, but CNS relapse remained common [125,126], consistent with real-world studies showing high rates of distant metastases, particularly brain metastases, in Stage III *EGFR*-mutated NSCLC [127]. The ASCENT trial, which used neoadjuvant afatinib followed by chemoradiotherapy (CRT) with or without surgery and possible adjuvant afatinib, again reported high rates of CNS-only relapses [128]. Given its superior systemic and CNS efficacy in both metastatic and resected early-stage disease [7,15], osimertinib therefore became the ideal post-CRT strategy to eradicate CNS micrometastases. In the LAURA trial, osimertinib after definitive CRT markedly improved PFS versus placebo (median, 39.1 months [95% CI, 31.5–NC] vs. 5.6 months [95% CI, 3.7–7.4]; HR, 0.16; 95% CI, 0.10–0.24; *p* < 0.001). The 12-month cumulative incidence of CNS progression was lower with osimertinib than with placebo (9% vs. 36%). Median CNS PFS was not reached with osimertinib (95% CI, NC–NC) versus 14.9 months with placebo (95% CI, 7.4–NC), resulting in an HR of 0.17 (95% CI, 0.09–0.32; nominal *p* < 0.001), which was almost identical to the overall PFS HR. Altogether, these findings suggest that improved CNS protection from osimertinib is likely a major contributing factor to the survival benefit observed after CRT [18].

Still, key questions remain: do all patients require lifelong osimertinib, and can we identify lower-risk patients for treatment deintensification? Can osimertinib be introduced earlier, prior to CRT, to further improve outcomes for patients at risk of early disease progression during CRT? The NEOLA trial addresses this question, using 8 weeks of induction osimertinib followed by platinum-based sequential or concurrent CRT, then maintenance osimertinib. The primary endpoint of the study is 12-month PFS. Interestingly, in a pre-planned interim analysis, induction osimertinib reduced planning target volumes by a mean of 27% (range, −69% to 8%), with 77% of patients demonstrating an objective response [129]. Given the concern of radiation pneumonitis (RP) leading to dose interruption and discontinuation in the osimertinib arm in 32% and 5% of patients, respectively, in the LAURA trial [18], whether induction osimertinib can optimize lung and heart dosimetry, thereby lowering RP rates, is an important question, and long-term safety data from NEOLA are eagerly anticipated.

### 5.3. The Evolving Role of Consolidative Local Therapy in Metastatic Disease

For patients with metastatic *EGFR*-mutated NSCLC, randomized data supporting local consolidative therapy (LCT) initially came from studies using earlier-generation TKIs. The phase III SINDAS trial showed that 5-fraction RT added to first-generation TKI improved both PFS (HR, 0.22; 95% CI, 0.17–0.46; *p* < 0.001) and OS (HR, 0.44; 95% CI, 0.28–0.68; *p* < 0.001) [130]. The Northern Radiation Oncology Group of China trial (NROG-002) found that fractionated thoracic and regional lymph node RT with TKI improved PFS (median, 17.1 months vs. 10.6 months; HR, 0.57; 95% CI, 0.34–0.84; *p* = 0.004) and OS (median, 34.4 months vs. 26.2 months; HR, 0.62; 95% CI, 0.41–0.96; *p* = 0.029) compared to TKI alone, in patients with no more than three synchronous metastatic sites. This benefit was primarily driven by an improvement in locoregional PFS (HR, 0.345; *p* < 0.001), but was accompanied by a higher rate of severe treatment-related adverse events (TRAEs), including radiation esophagitis and RP [131]. More recently, the randomized phase II NORTHSTAR trial extended this question of LCT to osimertinib. Despite a significant proportion of patients having polymetastatic disease, adding LCT improved PFS (median, 25.3 months vs. 17.5 months; HR, 0.66; 95% CI, 0.50–0.87; *p* = 0.025), with benefit reported across subgroups, including patients with polymetastatic disease and both exon 19 deletion and exon 21 L858R mutation subtypes. Interestingly, patients with polymetastatic disease who underwent complete LCT had a median PFS of 27.9 months, compared with 14.5 months with partial LCT. Crucially, there were no grade 4–5 LCT-related TRAEs. Taken together, the NORTHSTAR study supports the integration of LCT with third-generation TKIs, although optimal patient selection and the selection of optimal local treatment remain unresolved [132].

### 5.4. Safety Considerations in Combining EGFR TKIs and RT

Safety remains an important consideration when combining RT with *EGFR* TKIs. A retrospective multicenter study found more toxicity when *EGFR* TKIs were continued during RT, although severe acute or late toxicity did not differ significantly between interrupting and continuing TKIs [133]. A European Organization for Research and Treatment of Cancer (EORTC)-European Society for Radiotherapy and Oncology (ESTRO) OligoCare consortium consensus review concluded that RT dose and fractionation need not change with *EGFR* TKI, but no consensus was reached on whether TKI should be taken on RT days and how long any interruption should be [134]. Until more safety data are available, careful patient selection and individualized RT delivery will be essential to maximize tolerability and minimize harm when integrating RT with *EGFR* TKIs.

## 6. Challenging Clinical Situations and Future Directions

### 6.1. Small-Cell Transformed EGFR-Mutant NSCLC

Small-cell transformation is a recognized acquired resistance mechanism, especially post-third-generation *EGFR* TKI, and is associated with concurrent *TP53* and *RB1* alterations [46,135]. De novo small-cell lung cancer (SCLC) with *EGFR* mutations is also a recognized entity, albeit extremely rare, with a predilection for non-smoking females, sharing immunohistochemical similarities with conventional SCLCs [136]. Both de novo and acquired small cell transformation in *EGFR*-mutant lung cancer portend extremely poor prognosis, with multiple retrospective series estimating median overall survival of approximately a year or less [135,136,137,138]. Although immune checkpoint inhibitor (ICI) plus chemotherapy is the standard first-line treatment for conventional SCLC, the efficacy of this approach in patients with *EGFR*-mutant SCLC is less clear, with conflicting results from real-world studies, although definitive conclusions are limited by small sample sizes and retrospective data [137,139]. Given the immunosuppressive tumor microenvironment and lack of benefit of ICI-based approaches in *EGFR*-mutant NSCLC, it is unsurprising that *EGFR*-mutant SCLC has a distinct paradigm from conventional SCLC [140,141]. Yet, despite these tumors retaining the driver *EGFR* mutation post-SCLC transformation, the role of continuing *EGFR* TKI in combination with chemotherapy is also controversial, as retrospective series have not demonstrated improvement in survival outcomes over chemotherapy alone [137,139].

Given the emerging role of tarlatamab in conventional SCLC [142,143], coupled with increased delta-like ligand 3 (DLL3) expression after SCLC transformation in EGFR-mutant lung cancer [139,144], whether tarlatamab will be effective in this patient population is an interesting question. In a real-world series of 16 patients from the USA, only one patient achieved an objective response (7%), and this patient notably received osimertinib plus tarlatamab [145]. The evidence for osimertinib plus tarlatamab is only limited to case reports at present [144,145], which warrants prospective evaluation in addition to the role of DLL3 enrichment as a selection biomarker. Tarlatamab in combination with etoposide, carboplatin and atezolizumab is currently being examined in patients with SCLC post-EGFR TKI (NCT06830694), which will hopefully provide more treatment options in this poor prognostic patient population.

### 6.2. The Conundrum of Multiple Options and the Role of Biomarker Selection

Biomarker-selected approaches have proven challenging in the *EGFR*-mutant NSCLC clinical trial landscape, given the smaller patient population and need for molecular testing, with up to 18% of patients dropping out of the trial despite testing biomarker-positive [40]. Consequently, biomarker-agnostic strategies such as MARIPOSA-2 have gained traction worldwide and are currently preferred treatment options post-osimertinib, according to NCCN, ASCO, and ESMO Living Guidelines [32,146,147]. However, with the increasing number of competing treatment options, biomarkers are urgently needed to rationalize treatment decisions. While *MET* amplification is generally considered a robust biomarker for predicting the efficacy of *MET* TKI in combination with *EGFR* TKI, the role of immunohistochemistry (IHC)-based biomarkers is less clear [54]. For example, while c-*MET* IHC 3+ was initially proposed as a biomarker to select patients for amivantamab plus lazertinib post-osimertinib [148], subsequent analyses from CHRYSALIS-2 reported that the differences in ORRs between *MET* IHC-positive and IHC-negative tumors did not reach statistical significance to support the role of c-*MET* IHC as a selection biomarker for amivantamab with/without lazertinib [149]. Although *HER2* IHC 3+ has received tumor-agnostic approval for trastuzumab deruxtecan from the FDA, data supporting efficacy in *HER2* IHC 3+ NSCLC remain limited to 17 patients [150]. Notably, exploratory analyses from DESTINY-Lung03 observed that patients treated with prior *EGFR* TKI achieve higher ORR as compared to those without prior *EGFR* TKI, suggesting that patients with *EGFR*-mutant NSCLC with *HER2*-overexpression may benefit more from trastuzumab deruxtecan [151]. Beyond IHC, novel biomarkers, such as *TROP2* normalized membrane ratio (NMR) status assessed by quantitative continuous scoring (QCS), have demonstrated promise in selecting patients for Dato-DXd [152,153]. However, *TROP2* NMR status was not associated with improved clinical outcomes in patients with *EGFR*-mutant NSCLC treated with Dato-DXd with/without osimertinib [154]. Collectively, these observations suggest that biomarker testing strategies may need to be tailored to oncogene subsets within NSCLC rather than adopting a one-size-fits-all approach, and more research is needed to understand the implications of these biomarkers specific to *EGFR*-mutant NSCLC.

For clinical decision-making, biomarkers should be separated into predictive, prognostic, and exploratory categories. An example of a predictive biomarker would be *MET* amplification, which can be used to identify patients who may benefit from savolitinib plus osimertinib [31,41]. On the other hand, biomarkers such as ctDNA positivity, concurrent *TP53* mutations, and the presence of CNS or liver metastases are primarily prognostic and should not be used in isolation to select patients for upfront combination therapy. Exploratory biomarkers are those that have shown early efficacy signals but still require further validation, such as HER2 IHC, as discussed above.

Practical implementation is limited by assay and sampling constraints. Tissue biopsy permits histology, FISH, and protein assessment but is invasive and spatially limited. Plasma ctDNA can capture broader heterogeneity but may be falsely negative in low-shedding or CNS-predominant disease, underdetects copy-number changes such as *MET* amplification, and cannot diagnose transformation. MET definitions also vary across gene-copy, MET-to-CEP7 ratio, and IHC cutoffs. Prospective trials should therefore prespecify assays and thresholds and demonstrate that biomarker-guided treatment improves outcomes rather than merely correlating with prognosis.

ORCHARD provides an operational example of prospective biomarker-directed testing after first-line osimertinib. Post-progression tissue profiling allocated patients to biomarker-matched osimertinib combinations, non-biomarker-selected therapy, or an observational cohort for transformation or resistance mechanisms managed outside the platform [155]. Biological plausibility did not invariably translate into clinical utility: osimertinib plus necitumumab for *EGFR* amplification or selected secondary EGFR alterations yielded an objective response rate of 11% and a median PFS of 4.0 months, and further development was not supported [156]. By contrast, a non-biomarker-restricted osimertinib plus datopotamab deruxtecan module showed responses both with and without identifiable baseline resistance alterations [157]. Because the modules were non-randomized, they establish prospective activity within defined strata rather than proving that biomarker-based allocation improves outcomes over an unselected strategy.

### 6.3. Fourth-Generation EGFR Inhibitors

The term ‘fourth-generation EGFR inhibitor’ describes an investigational strategy rather than a regulatory class. These mutant-selective agents are designed mainly to inhibit EGFR C797S/C797X and compound resistance while sparing wild-type EGFR; some are also optimized for CNS penetration. None of the fourth-generation compounds has received regulatory approval to date, and evidence is limited to early-phase trials. Silevertinib (BDTX-1535) produced an objective response rate of 36.4% in a small single-arm C797S cohort and remains under clinical evaluation [158,159].

Development attrition illustrates the challenge of this class: the BBT-176 study was terminated after showing only early clinical signals [160,161]. Allelic context, coexisting bypass mechanisms, CNS activity, and tolerability, will determine whether fourth-generation inhibitors are used alone, combined with osimertinib, or restricted to molecularly selected trials. Enrollment in a genotype-matched clinical trial remains the preferred approach for EGFR C797X-mediated resistance.

BLU-945 is an oral, reversible, wild-type-EGFR-sparing inhibitor designed to target activating EGFR mutations and T790M/C797X resistance mutations. Preclinical studies demonstrated activity in osimertinib-resistant models as monotherapy and in combination with osimertinib [162,163]. In the phase I SYMPHONY analysis, BLU-945 produced dose-dependent suppression of circulating T790M, C797S, and L858R alleles and tumor reductions, including partial responses, but the findings remained preliminary, and the study was subsequently terminated by sponsor decision, unrelated to safety [164,165]. Notably, further global investment in the clinical development of BLU-945 was discontinued in 2024 despite promising early signals, partly because of the rapidly evolving treatment landscape, highlighting the challenges of drug development in this space [166].

BH-30643 has been described as a novel macrocyclic, non-covalent, mutant-selective OMNI-EGFR inhibitor designed to inhibit diverse activating and resistance mutations, including C797S with or without T790M, while largely sparing wild-type EGFR. In the first-in-human SOLARA dose-escalation analysis, 10 of 17 patients with C797S-positive disease who had an on-treatment scan achieved a confirmed or ongoing partial response, with activity also reported in the CNS. These results are promising but preliminary because they derive from an early-phase, non-randomized selected scan-evaluable subgroup [167].

### 6.4. Emerging Nucleic-Acid and Extracellular-Vesicle Delivery Platforms

Beyond protein therapeutics, nucleic-acid delivery platforms seek to silence oncogenic EGFR alleles or deliver regulatory RNA selectively to tumor cells. Mutation-selective antisense oligonucleotides packaged in red-blood-cell-derived extracellular vesicles reduced mutant EGFR expression and tumor growth in preclinical TKI-resistant models, and EGFR-aptamer-decorated exosomes carrying survivin siRNA enhanced uptake and suppressed EGFR-expressing NSCLC xenografts [168,169].

An inhalable p53 mRNA/lipid-liquid nanoplatform has shown preclinical antitumor activity in NSCLC models, but it is not EGFR-specific and has not established therapeutic efficacy in patients with *EGFR*-mutant NSCLC [170]. These platforms should therefore be considered preclinical or proof-of-concept. Major barriers include scalable GMP manufacture, batch consistency, payload loading, biodistribution, endosomal escape, innate immune activation, and mutation-selective tumor delivery.

### 6.5. AI, Clinicogenomic Data, and Multi-Omics for Personalized Treatment

The most credible near-term role of artificial intelligence (AI) is to complement rather than replace molecular testing. Deep-learning models can extract EGFR-associated features from histology, and multimodal models can integrate imaging and clinical features to estimate osimertinib resistance, but reported performance is largely retrospective and is insufficient to direct EGFR-targeted therapy without validated tissue or plasma genotyping [171,172].

Acknowledging these limitations, the implementation of multi-omic data in clinical practice will necessitate the use of AI. For example, integrating data from different sources, including CT scans, pathology images, and ctDNA results, with routine clinicopathological features to personalize treatment options or predict outcomes such as recurrence risk will require specialized AI models harnessing big-data analytics [171,172]. With the increasing number of treatment options, integrating multi-omic data to personalize treatment decisions is a pressing need [173,174]. Furthermore, existing technologies such as MRD do not achieve sufficient sensitivity to precisely inform the optimum duration of adjuvant *EGFR* TKI therapy [78] and may necessitate incorporating other parameters such as radiomics, although these approaches require prospective validation. To that end, collaboration between data scientists and clinicians is necessary to translate such efforts into clinical practice.

## 7. Conclusions

In conclusion, strides have been made in the management of *EGFR*-mutant NSCLC across the stages, yet numerous clinical challenges and unanswered questions remain. Urgent research priorities include biomarker development to rationalize treatment choices amid the expanding number of competing options, which must also be feasible in routine clinical practice, and optimizing toxicity management with the increased adoption of combinatorial strategies and novel therapeutics. From a healthcare economics perspective, assessing the cost-effectiveness of novel treatment strategies may become increasingly important in reimbursement decisions from global regulatory authorities. To that end, fostering collaboration between clinicians, payers, and industry stakeholders will be essential to ensure that therapeutic advances translate into equitable, sustainable, and accessible care for all patients with *EGFR*-mutant NSCLC.

## Data Availability

No new data were created or analyzed in this study. Data sharing is not applicable to this article.
